# Endoscopic submucosal dissection of gastric fundus subepithelial tumors originating from the muscularis propria

**DOI:** 10.3892/etm.2013.1181

**Published:** 2013-06-25

**Authors:** LEI LI, FENG WANG, BO WU, QINGCAI WANG, CHANGHUI WANG, JIYONG LIU

**Affiliations:** 1 Department of Gastroenterology, Shandong Provincial Hospital, Shandong University, Jinan, Shandong 250021;; 2 Department of General Surgery, Dezhou People’s Hospital, Dezhou, Shandong 253014;; 3 Department of General Surgery, Taian Eighty-eight Hospital, Taian, Shandong 271000;; 4 Department of Gastroenterology, Taian Central Hospital, Taian, Shandong 271000, P.R. China

**Keywords:** endoscopic submuscosal dissection, gastric fundus, muscularis propria

## Abstract

Endoscopic resection of gastric subepithelial tumors (SETs) carries a high risk of perforation, particularly for tumors located at the gastric fundus and originating from the muscularis propria. Based on our experience with endoscopic submucosal dissection (ESD) and a novel endoscopic device, namely the ‘Resolution clip’ for the endoscopic closure of iatrogenic upper gastrointestinal (upper GI) perforations, we evaluated the clinical feasibility and safety of ESD for gastric fundus subepithelial tumors originating from the muscularis propria. In this prospective study, 11 consecutive patients who presented with gastric SETs ≤3 cm in diameter were enrolled. Regardless of whether perforation occurred, the gastric wall defect was closed with clips. The patients were followed up after the surgery. Endoscopic resection was successfully performed in 10 patients; however, in one patient a pure endoscopic approach was impossible as the lesion was severely adhered to surrounding tissue, and a switch to laparoscopic wedge resection was necessary. The mean resected tumor size was 18.8×17.2 mm and the mean surgery time of the 10 patients with ESD was 81 min (range 45–130 min). Histological diagnosis was gastrointestinal stromal tumor (GIST) in eight lesions [very low risk according to the National Institutes of Health (NIH) risk classification] and leiomyoma in three lesions. Perforation occurred in 3/10 patients. Gastric closure with the Resolution clips was performed successfully in all cases. Early post-ESD bleeding (EPEB) occurred in one patient. Basic ferric sulfate solution was sprayed during the upper GI endoscopy examination and the bleeding stopped. No complications occurred and the follow-up was unremarkable. In this early study, ESD using the Resolution clip was demonstrated to be a feasible and minimally invasive treatment for gastric fundus subepithelial tumors originating from the muscularis propria.

## Introduction

Gastric subepithelial tumors (SETs) have a prevalence of ∼0.4% and are usually detected incidentally during upper gastrointestinal (GI) endoscopy (1). The SET appears as a mass, bulge or impression covered by normal epithelium with a protrusion to the inside (intramural tumor) or outside (extramural tumor) of the gastric wall (2). The majority of the tumors are benign, but potentially and overtly malignant lesions should not be neglected (3). According to current guidelines, large (diameter, >3 cm) or symptomatic SETs require surgery due to their malignant potential. However, the detection of a small SET (diameter, ≤3 cm) presents diagnostic and therapeutic dilemmas. The differential diagnosis is long and includes nonneoplastic lesions, benign neoplasms and potentially and overtly malignant tumors. Small asymptomatic SETs require periodic follow-up by endoscopy, particularly by endoscopic ultrasound (EUS) examinations (4,5). However, the definite discrimination of benign lesions from malignant lesions may only be achieved by histopathological examination (6). Previous studies have shown that a standard endoscopic forceps biopsy and EUS-guided fine needle aspiration (EUS-FINE) typically fails to obtain material adequate for diagnosis (7–9). Therefore, an accurate histopathological diagnosis may only be performed by removal of the SET.

Traditionally, surgical approaches for removal include open and laparoscopic or thoracoscopic surgery. Endoscopic methods, including snare polypectomy, band ligation and endoscopic submucosal dissection (ESD), have been used for the removal of GI SETs, but their use has generally been restricted to tumors located in the muscularis mucosae or submucosal layers (10,11). En bloc resection of subepithelial tumors originating from the muscularis propria layer using ESD remains problematic (12). The location of the tumor is a point of concern when performing this procedure. Due to the knife vertically orienting to the muscularis propria layer as a result of retroflexion of the endoscope, when the tumor is in the fundus the dissection is more challenging and more-time is taken for the resection than when the tumor is in the body or the antrum. The risks are also greater for resection of a tumor in the fundus than for those in other locations. Iatrogenic perforation and its inadequate closure are reported to be the major complications of this procedure (13,14). New techniques, such as the use of the Resolution clip, have been developed that enable secure closure of iatrogenic perforations and have already been successfully used in clinical practice in Shandong Provincial Hospital (Jinan, China), Taian Central Hospital (Taian, China) and Dezhou People’s Hospital (Dezhou, China).

The aim of this study was to evaluate the feasibility of resection of small gastric SETs using the ESD technique followed by closure of the gastric wall using Resolution clips.

## Patients and methods

### Study design and study population

The study protocol was approved by the Ethics Committees of Shandong Provincial Hospital, Taian Central Hospital and Dezhou People’s Hospital. In this retrospective single-center analysis, 11 consecutive patients (5 men, 6 women; median age 59.3 years, range 33–78) with gastric SETs were enrolled between October 2011 and December 2012 in Shandong Provincial Hospital, Jinan, China. At first, EUS was performed with a radial-scanning echo endoscope (GIF-T140; Olympus Optical Co. Ltd., Tokyo, Japan) to determine the size, layer of origin, margin and growth pattern of the SETs. The included patients met the following criteria: i) age >18 years; ii) maximum size was measured by the EUS examination before ESD between 1 and 3 cm as determined by EUS; iii) intramural growth assessed by EUS; iv) tumors originated from the muscularis propria; v) tumors located at the gastric fundus. Written informed consent to undergo ESD was obtained from all included patients after detailed spoken and written explanations were provided concerning the ESD procedure and other possible treatment options. Exclusion criteria, as used in a previous study, were as follows: i) no consent from the patient; ii) American Society of Anesthesiologists’ (ASA) class IV or V; iii) pregnancy; iv) disorders of blood coagulation; v) contraindications for endoscopy; vi) intramural or extramural large blood vessels within the resection area detected by EUS (15).

### Study apparatus

The main apparatuses used include an GIF-T140 endoscope, a double-channel upper GI endoscope (GIF-H260), a double-bending double-channel upper GI endoscope (GIF-2T260M), a transparent hood (D-201-11802), an insulated-tip knife 2 (IT-knife 2, KD-611L), a dural knife (KD-650L), Coagrasper hemostatic forceps (FD-410LR), an injection needle (NM-4L-1) and a snare (SD-9L-1) all from Olympus Optical Co. Ltd., Resolution clips (Boston Scientific, Natick, MA, USA), endoclips (HX-600-135; Olympus Optical Co. Ltd.), a high-frequency generator (ICC200; Erbe Elektromedizin GmbH, Tübingen, Germany), an argon plasma coagulation (APC) unit (APC300; Erbe Elektromedizin GmbH) and an auxiliary water jet (GIF-Q260J; Olympus Optical Co. Ltd.).

### Study procedures

The surgery was performed in the operating theater with the patient under mechanically ventilated general anesthesia and electrocardiographic monitoring.

**Figure 1.**
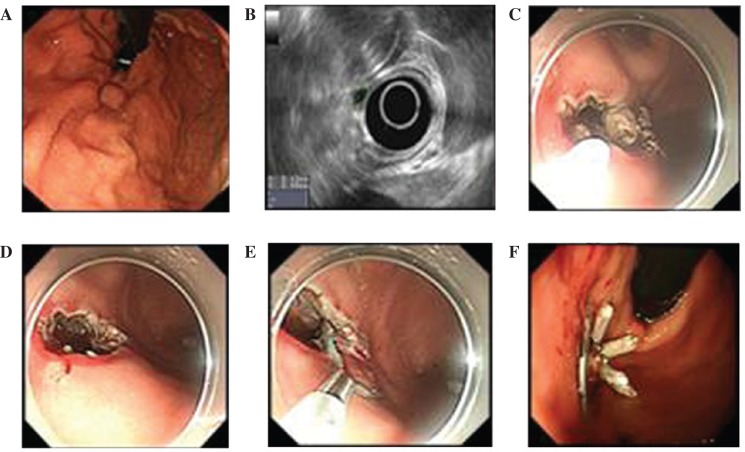
The ESD procedure. (A) Submucosal tumor identified in the gastric fundus by endoscopy. (B) Endoscopic ultrasonographic evaluation of the tumor originating from muscularis propria. (C) The tumor was then dissected from the muscle. (D) The defect and perforation of the gastric wall. (E) Closure with Resolution clip. (F) An adequate closure of the gastric wall perforation using clips.

The procedure (Fig. 1) began by marking the lesion margins with APC. The tissue at the proximal end of the subepithelial lesion was injected with 1–2 ml of a mixture prepared by diluting epinephrine (1 mg) and 0.8% indigo carmine (2 ml) dye in 0.9% saline solution (500 ml) to create a submucosal liquid pool. A precut of 3–5 mm was made at the injection site using a dural knife with the electrosurgical generator in the 30 W EndoCut mode. The IT-knife 2 was placed at the initial incision to dissect the tissue and create a circular incision around the lesion. When the submucosa was completely separated from the tumor, the underlying muscularis propria was dissected away to lift the tumor. During the dissection, it was necessary to coagulate all visible vessels in the muscular and submucosal layers and stop any bleeding using a forceps coagrasper or by APC prior to the next step of the resection. Since the tumor was located at the fundus, the final step of the dissection was performed using the technique of polypectomy by employing an electrocautery snare using blended electrosurgical current. All tumors were retrieved by a net. The large defect of the gastric wall following resection was closed completely with clips. Resolution clips were used to close the defect or perforation first in order to narrow the leaks. Endoclips were used for the closure of the remaining small leaks. At the end of the procedure, a leakage test was performed with methylene blue dye. Complete resection was defined as the absence of any tumor remnant when viewed endoscopically following resection. The patients were given GI decompression and remained *nil per os* for three days with parenteral alimentation and proton pump inhibitor treatment.

### Pathological examination

The removed tumors were paraffin-embedded and sectioned for histopathological and immunohistochemical analysis. Staining was carried out with hematoxylin and eosin (H&E). Additionally, immunohistochemical staining was performed on paraffin-embedded tissue sections. Positive reactions for DOG-1 or CD34 were considered diagnostic of a gastrointestinal stromal tumor (GIST) and in cases where a GIST was suspected, the analysis included a mitotic count under a high-power field (HPF) in order to determine the malignant potential according to the classification of Miettinen and Lasota (5). Immunohistochemical analysis of CD117, smooth muscle actin (SMA), desmin, S-100 and Ki67 markers was also performed to classify the tumor subtype. Resection of the tumor was regarded as complete when dissection margins were negative for tumor tissue (R0 resection) and regarded as incomplete when there were positive margins (R1 resection) (17). Achievement of R0 resection for gastric SETs with subsequent adequate closure of the gastric wall was the target of the surgery and study.

### Patient follow-up

The included patients were scheduled for follow-up by telephone interview or at an outpatient visit 2 weeks after the procedure, and by standard upper GI endoscopy 8 weeks after the procedure. The interval between surveillance examinations was extended to 6 months for leiomyomas and 3 months for GISTs based on the results of histopathological evaluation.

## Results

The characteristics of the 11 patients included in the current study and their treatment outcomes are summarized in Table I and in Fig. 1. All lesions were located at the fundus and originated from the muscularis propria. Complete resection was achieved in 10 of 11 lesions (90.9%). A switch to laparoscopic wedge resection was necessary in one patient in whom the tumor was attached to surrounding tissue (Table I). The mean resected tumor size was 18.8×17.2 mm, and the mean operation time of the 10 patients with ESD was 81 min (range 45–130 min).

Gastric perforation occurred in 3/11 patients (27.2%). All perforations and defects were closed successfully by endoscopic techniques using clips without surgical treatment (Table I; Fig. 1). Early post-ESD bleeding (EPEB) occurred in one patient. Basic ferric sulfate solution was sprayed during the upper GI endoscopy and the bleeding stopped (Table I).

All 10 tumors that were removed endoscopically showed macroscopically complete resection; R0 resection was achieved with basal tumor-free margins microscopically. Eight patients (72.7%) had GISTs. The HPF mitotic counts of all resected tumors were low (<5 mitosis/50 HPFs). All GISTs were completely resected. During follow-up, peritonitis and abdominal abscess were not observed in the patients.

## Discussion

Upper-GI SETs are often discovered incidentally during routine upper GI endoscopic examination in the clinic. The recommended management strategy includes periodic follow-up endoscopy and EUS (18). However, the optimum method and interval of follow-up of SETs have not yet been precisely established. Indefinite follow-up examinations without definite diagnosis may cause an enormous emotional strain on patients (19). In addition, accurate diagnosis is essential since a subset of these lesions do have malignant potential, particularly GISTs originating from the muscularis propria (20). ESDs are performed to remove the whole tumor, which may be analyzed histopathologically. Despite the development and modification of endoscopic resection by ESD, recent studies have reported that gastric SETs originating from the muscularis propria layer may be successfully enucleated by endoscopy (13,21). However, the complete endoscopic resection of gastric fundus SETs that originate from the muscularis propria is more challenging than that of tumors from other locations and layers in the stomach (13,21,22). The reasons may be as follows: i) The gastric fundus is in the upper portion of the stomach and the operation requires retroflexion of the endoscope. ii) The muscularis propria is a deep layer of gastric wall and adjacent to the serosal layer. For this reason, endoscopic resection has a higher rate of perforation than the same procedure when used for the treatment of lesions located in other gastric areas.

In addition to a double-channel upper GI endoscope (GIF-H260; Olympus), a double-bending double-channel upper GI endoscope (GIF-2T260M; Olympus) was used in our ESD procedure. By using GIF-2T260M, we were able to focus on the lesion more accurately and avoid misjudgment and mishandling. In the present study, we performed ESD in 11 patients and complete endoscopic resection of 10 upper-GI SETs that originated from the muscularis propria. The unsuccessful case was a patient who had a tumor severely adhering to surrounding tissue. The complete resection rate was higher than reported by Shim and Jung (16) and similar to that in a study by Liu *et al* (13). However, in the study conducted by Liu *et al*, only two lesions were located at the fundus.

The perforation rate (27.2%) was higher in the current study than that in a previous study on ESD by Tanaka *et al* (23). This may due to the location and origin of the lesions. Several methods for the closure of gastric endoscopic full-thickness resection have been described in a preclinical and clinical setting (24–27), but thus far the majority of these methods are technically challenging, require specialized equipment and are thus limited with respect to reproducibility and widespread applicability (15,24,25). In the present study, the perforations were closed by clips. The defects following surgery were also closed by clips to prevent delayed perforation. The two types of clips used were Resolution clips from Boston Scientific and endoclips from Olympus Optical Co. Ltd. The diameter of the Resolution clip is ∼13 mm, which is larger than that of the endoclip. To handle the defect or perforation, we first used the larger clip to minimize the leakage and then used the endoclip to make a complete closure. All the defects and perforations were closed successfully.

EPEB occurred in one patient. The patient had a reduction in hemoglobin level of 3 g/dl within 16 h after surgery. Basic ferric sulfate solution was sprayed during the upper GI endoscopy and the bleeding stopped. EPEB is a common ESD-associated complication with the occurrence of clinical symptoms and laboratory changes (hemoglobin reduction >2 g/dl) that indicates GI bleeding within 48 h of the ESD (28). In a large-scale study, the rates of bleeding differed significantly in relation to the location of the lesion, origin of the lesions, presence of a scar, histological type and ESD time (29).

In the current study the majority of the resected SETs were GISTs with very low risk and the others were leiomyomas. However, the malignant potential of a GIST may not be reliably determined in advance by either endoscopic or endosonographic techniques (29). Alternative endosonographic surveillance may delay the diagnosis of malignancy and cause strain in many patients. Therefore, endoscopic resection appears an advisable, less invasive therapeutic option, although over-treatment of benign lesions may occur. We observed that 72.7% of the resected SETs were GISTs, which was similar to the findings of a previous study (30). Current guidelines of the National Comprehensive Cancer Network recommend that all GISTs >2 cm should be resected and that incidentally encountered GISTs <2 cm may be either followed up or resected (31). However, there remain certain contradictions concerning the guideline (32). R0 resection of all suspected lesions appears advisable. Local resection with gross negative margins and without lymph node resection is considered a curative approach since GISTs rarely have lymph node metastasis. As the defects and perforations may be closed completely by clips, we achieved R0 resection of all GISTs.

Our study has certain limitations. Firstly, certain new techniques, such as submucosal tunneling, may be evaluated for SETs located at the fundus next to the cardia. Secondly, more patients are required for further studies.

In our opinion, a classic ESD technique using clips for the dissection of small gastric fundus SETs from the deep muscularis propria layer is feasible and easy to conduct. Perforations that occur following full-thickness resection may be adequately managed by clips.

## Figures and Tables

**Table I.** Clinicopathologic characteristics of patients in this study and treatment outcomes.

Patient no.	Age (years)	Gender	Tumor size (mm)	Procedure time (min)	Complete resection/complication	Pathology findings	Follow up time (weeks/recurrence)
1	59	F	15×15	95	Yes	Leiomyoma, SMA+, Desmin+, CD117-, Dog-, S-100-, Ki67<1%	45/no
2	57	F	20×18	100	Yes	GIST, SMA-, Desmin-, CD117+, Dog+, S-100-, Ki67=2%, mitosis/HPF=5/50 HPF	30/no
3	67	F	12×10	78	Yes	GIST, SMA-, Desmin-, CD117+, Dog+, S-100-, Ki67=2%, mitosis/HPF=5/50 HPF	35/no
4	60	M	15×9	62	Yes	Leiomyoma, SMA+, Desmin+, CD117-, Dog-, S-100-, Ki67<1%	25/no
5	48	M	25×15	110	Yes/perforation	GIST, SMA-, Desmin-, CD117+, Dog+, S-100-, Ki67=2%, mitosis/HPF=5/50 HPF	25/no
6	58	M	12×12	65	Yes	GIST, SMA-, Desmin-, CD117+, Dog+, S-100-, Ki67=2%, mitosis/HPF=5/50 HPF	30/no
7	53	M	30×28	130	Yes/perforation	GIST, SMA-, Desmin-, CD117+, Dog+, S-100-, Ki67=2%, mitosis/HPF=5/50 HPF	25/no
8	48	M	15×15	55	Yes/perforation, EPEB	GIST, SMA-, Desmin+, CD117+, Dog+, S-100-, Ki67=3%, mitosis/HPF=5/50 HPF	20/no
9	49	F	15×15	45	Yes	Leiomyoma, SMA+, Desmin+, CD117-, Dog-, S-100-, Ki67<1%	10/no
10	51	F	25×20	70	Yes	GIST, SMA-, Desmin+, CD117+, Dog+, S-100-, Ki67=3%, mitosis/HPF=5/50 HPF	10/no
11^a^	51	F	30×25	NA	NA	GIST, SMA-, Desmin+, CD117+, Dog+, S-100-, Ki67=3%, mitosis/HPF=5/50 HPF	NA

GIST, gastrointestinal stromal tumor; HPF, high-powered field; EPEB, early post-endoscopic submucosal dissection bleeding.

a The patient was switched to laparoscopic resection.
